# Practical Notes on Diagnosis and Treatment

**Published:** 1909-07-31

**Authors:** 


### PRACTICAL NOTES ON DIAGNOSIS AND TREATMENT.

**X-Rays in Leukæmia.**

Sufficient evidence has been brought forward in recent years to show that the *x*-rays are capable of exercising a beneficial action in many cases of myelogenous leukæmia, although the benefit may be only temporary. After a time the patient appears to acquire a sort of immunity to the rays and ceases to derive any benefit therefrom.—*Drs. Emanuel and Mackey.*

**Acute Synovitis of the Knee.**

It is a common practice to apply ice immediately, with a view, I suppose, to arresting the bleeding. That ice does at times to some degree relieve pain by rendering the part more or less numb, I have no doubt; but that it helps to avert the bleeding there is, I fancy, no evidence, and it is not always harmless.—*Sir W. H. Bennett.*

**Gunshot Wounds of the Abdomen.**

Experience has abundantly proved that laparotomy in the field is more dangerous than the gunshot wound for which it is performed. Surgeons of great eminence, who saw large numbers of gunshot wounds of the abdomen in South Africa, admitted that no amount of experience of these cases enabled them to form any opinion as to the course that a gunshot wound of the abdomen would follow.—*Major Spencer.*

**The Prognosis of Infantile Paralysis.**

Broadly speaking, any muscle which still reacts to faradism after ten days, even though still apparently paralysed, will probably recover. More than this, even a muscle whose faradic excitability is lost may recover to a considerable extent under appropriate treatment.—*Dr. Purves Stewart.*

